# Cervical Myelopathy Possibly Induced by Intraoperative Posture During Robot-Assisted Radical Prostatectomy

**DOI:** 10.7759/cureus.92081

**Published:** 2025-09-11

**Authors:** Yoshinori Maki

**Affiliations:** 1 Neurosurgery, Hikone Chuo Hospital, Hikone, JPN

**Keywords:** cervical laminoplasty, cervical myelopathy, complication, head-down position, robot-assisted radical prostatectomy

## Abstract

A lithotomy position is a standardized posture during robot-assisted radical prostatectomy, and several intraoperative complications related to this position have been described. However, cervical myelopathy possibly induced by this position during robot-assisted radical prostatectomy is seldom reported.

A 69-year-old man presented to the department of neurosurgery, complaining of sensory disturbance and motor weakness in the lower bilateral extremities. The patient underwent a robotic-assisted radical prostatectomy one month before. The patient was set in a lithotomy position for 4 hours and 25 minutes. Approximately two weeks after surgery, neurological deficits appeared and aggravated chronologically (finally equivalent to a Manual Muscle Testing of 1). The patient had not experienced any similar symptoms before prostatectomy. A blood examination was not suggestive of any inflammatory diseases. Cervical degenerative spondylosis was identified on whole spine magnetic resonance images. We considered that his neurological symptoms could be cervical myelopathy, possibly triggered by the lithotomy position during prostatectomy, because the posture and weight burden could have compressed the spinal cord. Cervical laminoplasty from C3 to C6 was performed to decompress the spinal cord. Two months after cervical laminoplasty, the motor weakness in the lower extremities recovered gradually, equivalent to a Manual Muscle Testing of 4, with rehabilitation therapy. After discharge from the hospital, the patient continues home visiting rehabilitation five months after surgery.

Cervical myelopathy may result from the lithotomy position during robot-assisted radical prostatectomy. Clinicians should be aware of this rare complication related to the intraprocedural posture in the urological field. Similar cases seem to be warranted to elucidate the mechanism of this rare postoperative complication.

## Introduction

Robotic-assisted radical prostatectomy is a standard surgical procedure for patients with localized prostate cancer [1]. This operation has advantages such as well-controlled intraoperative bleeding and postoperative pain, quick recovery, reduced admission period, and preserved urinary and sexual functions. Meanwhile, unfavorable complications were described in several fields: ophthalmic, otologic, cardiovascular, respiratory, renal, gastrointestinal, hepatobiliary vascular, musculoskeletal, and integumentary systems. Postoperative edematous change was also reported in the airway, conjunctiva, and subcutaneous tissue [2]. The peripheral nerve injuries related to the intraoperative posture during robotic-assisted radical prostatectomy seem relatively rare, with an incidence of 0.16% [3]. In the previous reports, the ulnar nerve, brachial plexus, and median nerve injuries were described in the upper extremities [2,4], while the lateral femoral cutaneous, common peroneal, obturator, and sciatic nerves were described in the lower extremities [2,4,5]. The pressure stress to the body related to the surgical posture is a risk [2]. The central nervous system is not exceptional, and postoperative cerebral edema has been reported in several reports [2,6-9]. Prolonged operation time, steep Trendelenburg, and high CO2 peritoneal concentration were considered as risk factors [2]. Postoperative agitation and depressed mental status can occur, and reintubation or extubation after the disappearance of cerebral edema should be indicated [2]. The utility of dexamethasone and diuretics for cerebral edema has also been described [2].

However, to our knowledge, little has been described concerning cervical myelopathy possibly induced by the intraprocedural posture of robot-assisted radical prostatectomy. Herein, we describe a rare case of cervical myelopathy following robot-assisted radical prostatectomy.

## Case presentation

A 69-year-old man underwent robot-assisted radical prostatectomy for prostate cancer. Under general anesthesia, the patient was placed in a lithotomy position for 4 hours and 25 minutes during the operation. The postoperative course was uneventful except for a COVID-19 infection eight days after surgery. The patient had a fever but recovered after the administration of encitrelvir fumarate. After we diagnosed the patient with COVID-19, the patient was instructed to stay in their room for a week to avoid spreading the infection. Following the infection, the patient complained of ambulation difficulty for long distances and sensory disturbance in the lower extremities, which had not bothered the patient before the prostatectomy. The patient was discharged from the hospital 16 days after surgery as the motor and sensory symptoms had resolved gradually and spontaneously. During the admission period, the patient was given antipyretic analgesics. No other infection occurred, nor were any medications that could induce motor weakness of the lower extremities, such as hypnotics, prescribed. No radiological examination was performed to avoid nosocomial infection. The patient did not wish for further examination, either, as the symptoms resolved. However, the neurological symptoms recurred 19 days after surgery, and the patient was referred to a neurologist. On neurological examination, the patient was conscious and alert. No deficits were observed in the cranial nerves. The upper extremities were powerful, but motor weakness was observed in the bilateral lower extremities (equivalent to a Manual Muscle Testing of 4+). No clumsiness was observed in the upper and lower extremities. However, hyperreflexia in the biceps, brachioradialis, and patellar tendons was accompanied by positive Babinski and Chaddock reflexes. The sense of vibration decreased in the lower extremities. The patient's ambulation exhibited a wide-based pattern, and he was unable to perform a tandem gait. These findings suggested impaired dorsal cord function. Followingly, bladder and rectum dysfunction also appeared. Blood and whole spine magnetic resonance imaging examinations were performed to rule out myelitis and cervical spondylosis. No inflammation findings were observed in the blood examination (Table 1).

**Table 1:** The result of blood sample examination No abnormal findings were observed in this blood examination.

	Results	Normal range
Total protein (g/dL)	6.3	6.6-8.1
Albumin (g/dL)	4.0	4.1-5.1
Total bilirubin (mg/dL)	0.4	0.4-1.5
Aspartate aminotransferase (U/L)	12	13-30
Alanine aminotransferase (U/L)	16	10-42
Lactate dehydrogenase (U/L)	85	124-222
Alkaline phosphatase (U/L)	40	38-113
γ-glutamyl transpeptidase (U/L)	24	13-64
Creatine kinase (U/L)	142	59-248
Amylase (U/L)	52	44-132
Blood urea nitrogen (mg/dL)	16.1	8-20
Creatinine (mg/dL)	0.80	0.65-1.07
Uric acid (mg/dL)	5.3	3.7-7.8
Inorganic phosphorus (mg/dL)	2.8	2.7-4.6
Calcium (mg/dL)	9.1	8.8-10.1
Sodium (mmol/L)	142.3	138-145
Potassium (mmol/L)	3.8	3.6-4.8
Chloride (mmol/L)	107.3	101-108
C-reactive protein (mg/dL)	0.04	< 0.14
Immunoglobulin G (mg/dL)	1012	861-1747
Immunoglobulin A (mg/dL)	203	93-393
Immunoglobulin M (mg/dL)	33	33-183
Complement C3 (mg/dL)	98	73-138
Complement C4 (mg/dL)	29	22-31
Antistreptolysin O titer (IU/ml)	20	< 160
Rheumatoid factor (IU/ml)	5.8	< 15.0
Thyroid-stimulating hormone (μIU/ml)	2.733	0.61-4.23
Free thyroxine (ng/dl)	1.1	0.9-1.8
Free triiodothyronine (pg/ml)	2.6	2.1-3.8
White blood cell count (×10^2^)	56	40-80
Red blood cell count (×10^4^)	384	450-550
Hemoglobin (g/dL)	12.5	13-17
Hematocrit (%)	37.2	37-53
Platelet count (×10^4^)	35.8	13-35
Mean corpuscular volume (fl)	96.9	83-99
Mean corpuscular hemoglobin (Pg)	32.6	27-35
Mean corpuscular hemoglobin concentration (%)	33.6	31-36
Neutrophil (%)	48.1	48-66
Eosinophil (%)	7.0	1-5
Basophil (%)	1.3	0-1
Lymphocytes (%)	35.2	25-45
Monocytes (%)	8.4	4-7
Neutrophil (/μl)	2680	2000-7500
Eosinophil (/μl)	390	0-400
Basophil (/μl)	70	0-150
Lymphocytes (/μl)	1960	1500-4000
Monocytes (/μl)	470	100-800
Prothrombin time (%)	102	70-140
Prothrombin time (seconds)	11.7	10.5-12.0
Prothrombin time-international normalized ratio	0.99	0.95-1.15
Activated partial thromboplastin time (seconds)	27.1	24-34
D-dimer (μg/ml)	1.1	< 1
Proteinase 3-anti-neutrophil cytoplasmic antibody (U/ml)	< 1.0	< 3.5
Myeloperoxidase anti-neutrophil cytoplasmic antibody (U/ml)	< 1.0	< 3.5
Anti-aquaporin 4 antibody	< 1.5	< 3.0
Vitamin B1 (ng/ml)	44.3	21.3-
Vitamin B12 (pg/ml)	850	233-914
Vitamin B6 (ng/ml)	6.0	3.6-12.9
Double-stranded DNA antibody E (IU/ml)	-	(-)
	0.7	< 10.0
Antinuclear antibody	< 40	< 40
Homogenous pattern	< 40	< 80
Speckled pattern	< 40	< 80
Nucleolar pattern	< 40	< 80
Centromere pattern	< 40	< 80
Peripheral pattern	-	< 80
Nuclear envelope type	< 40	< 80
Proliferating cell nuclear antigen type	< 40	< 80
Proliferating cell nuclear antigen-like type	< 40	< 80
Cytoplasmic type	-	(-)
Spindle fiber type	-	(-)
Anti-Golgi type	-	(-)
Granular type	< 40	-
Anti SS-A/Ro antibody	-	(-) or (±)
Index value	< 0.4	< 7.0
Anti SS-B/Ro antibody	-	(-) or (±)
Index value	< 0.4	< 7.0

**Figure 1:**
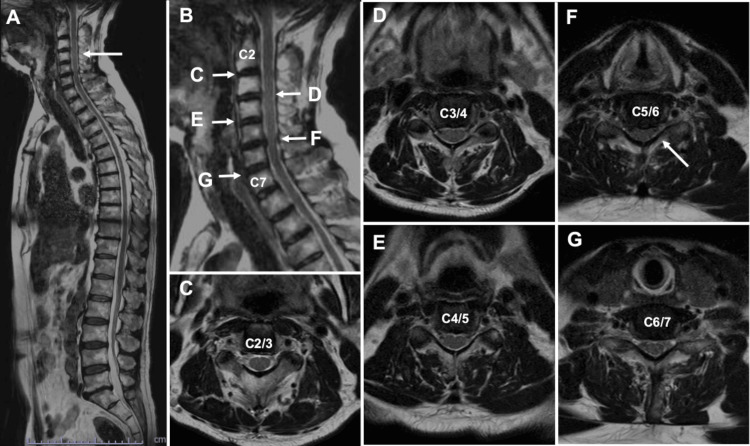
Initial whole spinal magnetic resonance images (A, B) Cervical degenerative spinal stenosis (white arrow) was observed, but no lesions were identified in the thoracic and lumbar levels. Hyperintensity lesions in the spinal cord were not observed, either. (C-G) The cervical spinal canal stenosis was observed from C2 to C6. The spinal canal was narrow, especially at the level of C5/6 (white arrow in F).

Cervical degenerative spondylosis was identified on magnetic resonance images, but no other findings suggestive of myelitis were observed (Figure 1).

**Figure 2:**
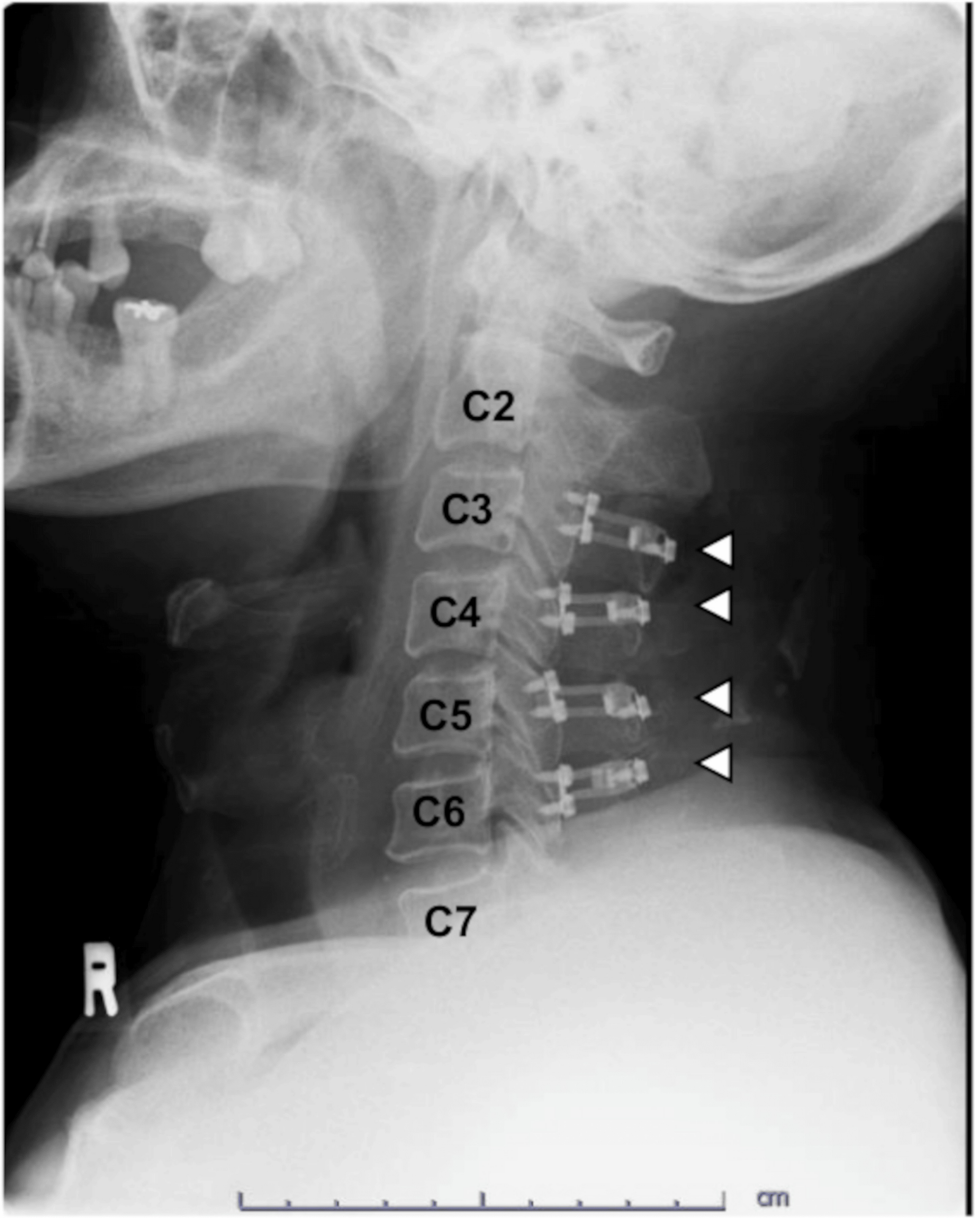
Postoperative cervical lateral X-ray image Cervical laminoplasty was performed from C3 to C6 levels. Spacers (white arrowheads) were observed.

Thereafter, the patient was referred to the neurosurgeon. The neurological deficits worsened gradually, and the sensory disturbance was observed below T10. The motor weakness of the lower extremities was also aggravated (equivalent to a Manual Muscle Testing of 3). Although the neurological symptoms fluctuated after robot-assisted radical prostatectomy, we thought that the neurological symptoms could have resulted from cervical degenerative spondylosis and from the spinal cord irritation related to the intraoperative posture. We planned cervical laminoplasty to decompress the cervical spinal cord two weeks after, but the patients became completely paraplegic several days before surgery. Rehabilitation therapy was soon initiated after cervical laminectomy from C3 to C6 (Figure 2).

The postoperative course was uneventful, and patients returned home after two months of rehabilitation therapy. His motor weakness of the lower extremities recovered to a Manual Muscle Testing of 4. However, his activities of daily living were not completely independent. Visiting home rehabilitation service was introduced to maintain the patients’ physical performance. The patient’s condition is stable five months after the operation.

## Discussion

Herein, we described a rare complication possibly related to a lithotomy position during robot-assisted radical prostatectomy. The cervical generative spondylosis was asymptomatic before surgery; however, sensory and motor neurological deficits appeared and gradually aggravated. Cervical laminoplasty was performed, and the patient’s neurological deficits were relieved after rehabilitation.

As for nervous system complications during robot-assisted radical prostatectomy, brain edema and peripheral nerve injuries in the upper and lower extremities were described [3-9]. The following relevant risk factors of these complications are postulated: over-abduction position, prolonged operation time, steep intraoperative posture, insufficient padding, and excess pressure against tissue [7,10-13]. In our case, the patient had cervical degenerative spondylosis, although the lesion was asymptomatic. As the mechanical stress to the spinal cord of elderly patients with degenerative cervical lesions can result in myelopathy [14], the stress on the cervical spinal cord should be minimized. We hypothesize that the stress to the cervical spinal cord, while the patient was set in a lithotomy position during robot-assisted radical prostatectomy, could have resulted in cervical myelopathy in our case. The postural and weight burden on the neck can increase due to the gravity effect during the lithotomy position, which seems to be a causative mechanism of postoperative cervical myelopathy. The impaired dorsal cord function observed in this patient is consistent with this hypothesis. To avoid this rare complication, the criteria of positioning during robot-assisted radical prostatectomy seem necessary. The restricted steepness of Trendelenburg to 30 degrees maximum is recommended to decrease the risk of cerebral edema [2]. Even though we do not have any apparent evidence related to cervical myelopathy related to robot-assisted radical prostatectomy, we should at least follow the aforementioned recommendation. Whether we need more restricted criteria to prevent cervical myelopathy after robot-assisted radical prostatectomy should also be evaluated with further similar reports in the future.

Before neurological deficits, our patient was positive for COVID-19. As a result, the patient’s daily activities were restricted so as not to spread the infection. This could have resulted in declined physical performance in a short period, as the patient complained of ambulation difficulty for long distances. COVID-19 can cause secondary neurological events, including cerebrovascular diseases, altered mental status, seizures, meningoencephalitis, and myelitis [15,16]. In our case, neurologists initially suspected that myelitis could have resulted in neurological deficits. Acute myelitis associated with COVID-19 infection has been reported in numerous cases [16-21]. The results of blood examinations varied, with or without any notable findings, including increased inflammatory markers [16-21]. However, abnormal findings, such as hyperintense lesions on T2-weighted images, were concurrently disclosed on spinal magnetic resonance imaging examinations [16,19-21]. A case of acute myelitis following COVID-19 infection was described by Águila-Gordo et al. In their case, degenerative changes in the intervertebral discs were observed at the C5-C6 level on magnetic resonance images. No hyperintensity lesions were identified in the whole spine levels, which seems quite similar to our case. Another case of acute myelitis related to COVID-19 was also reported without any radiological findings on spinal magnetic resonance images [18]. However, in those cases, an increased inflammatory reaction was observed in the blood examination. In addition, neither patient underwent surgery for prostate cancer before the acute myelitis [17,18]. Because we did not examine the patient’s cerebrospinal fluid, we could not completely exclude the possibility of a correlation between COVID-19 and neurological symptoms in our case. The laboratory and radiological findings related to acute myelitis related to COVID-19 can vary [16-21], and no management for the possible acute myelitis could have resulted in incomplete muscle recovery in the lower extremities.

We observed hyperreflexia in the patient before cervical laminoplasty, which seems inconsistent with the diagnosis of Guillain-Barré syndrome. In addition, no radiological findings on magnetic resonance images suggestive of vascular anomaly (i.e., arteriovenous malformation or fistula), such as flow voids with/without spinal cord edema, were observed in our case. Therefore, we thought that the cause of neurological symptoms could have resulted from the spinal cord stress during robot-assisted radical prostatectomy.

As this is a single case report, more similar cases should be reported to alert to the potential risk hidden in a lithotomy position during robot-assisted radical prostatectomy. Fluctuating neurological symptoms and concurrent COVID-19 infection may hinder the complete diagnosis of cervical myelopathy resulting from the intraoperative posture. Accumulation of similar cases can help clarify the mechanism of this manifestation.

## Conclusions

We reported a case of cervical myelopathy possibly induced by a lithotomy position during robotic-assisted radical prostatectomy. Though the patient had coincidentally contracted COVID-19, and the neurological deficits could have also been related to this disease, the blood sample and neurological examinations did not correspond to typical myelitis or Guillain-Barré syndrome. This postoperative complication seems to be scant in the literature; therefore, clinicians should be aware of this rare adverse event. To clarify the correlation of cervical myelopathy and a lithotomy position during robotic-assisted radical prostatectomy, more similar cases should be studied in further research.
